# Clinical and laboratory features in health care volunteers with inactivated SARS-CoV-2 vaccination

**DOI:** 10.55730/1300-0144.5684

**Published:** 2023-05-25

**Authors:** Zhijie LI, Yafang WAN, Lanlan XU, Wenjia ZHANG, Yu ZHANG, Pu LIAO

**Affiliations:** 1 Chongqing Medical University, Chongqing, China; 2 Chongqing Institute of Green and Intelligent Technology, Chinese Academy of Sciences, Chongqing, China; 3 Chongqing School, University of Chinese Academy of Sciences, Chongqing, China; 4 Department of Clinical Laboratory, Chongqing General Hospital, Chongqing, China

**Keywords:** COVID-19, healthcare, vaccination, CD19, IL-6, IL-2

## Abstract

**Background/aim:**

To better optimize the inactivated vaccine-induced immune response and improve vaccine protection efficiency, a preliminary study was conducted on the influencing factors of producing neutralizing antibody (NAb) titers against the inactivated coronavirus disease 2019 (COVID-19) vaccine.

**Materials and methods:**

A total of 91 health care volunteers were enrolled from the Immunology Division of the Laboratory Department of Chongqing General Hospital from February to March 2021. The study had a cross-sectional design. All of the volunteers were scheduled to receive a complete dose regimen of the inactivated severe acute respiratory syndrome coronavirus 2 (SARS-CoV-2) vaccine and the vaccination interval between 2 doses was 14 days. Clinical and laboratory features were collected for further analysis.

**Results:**

The NAb titers gradually increased after COVID-19 vaccination, and 72.53% (n = 66) of the volunteers had NAbs after the second dose. Eight variables, including CD16^+^CD56^+^ NK cell level before the first dose (HR = 0.94, p = 0.02), CD16^+^CD56^+^ NK cell level after the second dose (HR = 0.94, p = 0.03), interleukin (IL)-2 level before the first dose (HR = 2.09, p = 0.05), mean corpuscular volume (HR = 0.86, p = 0.02), serum urea level (HR = 0.69, p = 0.05), increment of CD19^+^ B cells (HR = 0.86, p = 0.03), increment of CD4^+^/CD8^+^ T cells (HR = 0.21, p = 0.03), and increment of the IL-6 level (HR = 0.75, p = 0.04) demonstrated a correlation with the NAb titers after COVID-19 vaccination. In the multivariate logistical regression analysis, the serum urea level (HR = 2.32, P = 0.03) and increment of CD19^+^ B cells (HR = 1.96, p = 0.03) were positively correlated with the NAb titers. The principal component analysis effectively distinguished the response after COVID-19 vaccination. The Pearson correlation analysis indicated that the CD19^+^ B cell level (r = 0.23, p < 0.001) and IL-2 (r = 0.24, p < 0.001) and IL-6 levels (r = 0.22, p < 0.001) were weakly positively correlated with the concentration of NAbs.

**Conclusion:**

The NAbs titers of the inactivated vaccines were positively correlated with the ratio of CD19^+^ B cell, IL-6, and IL-2 levels in the serum, which provide clinical guidance for inactivated SARS-CoV-2 vaccines.

## 1. Introduction

Coronavirus pneumonia (coronavirus disease 2019, COVID-19) infection is still prevalent worldwide [1]. Vaccination against COVID-19 is considered to be the most effective way to control the spread of the epidemic [2]. Presently, 12 types of COVID-19 vaccines are used worldwide. There are 7 types of COVID-19 vaccines marketed or approved for emergency use in China, 5 of which are inactivated [3]. Although 12 types of inactivated COVID-19 vaccines have been marketed, there are still few related studies focusing on inactivated vaccine-induced immune responses [4]. It is of great importance to determine the reactions to the vaccines and strengthen the formulation of immunization strategies for induced immune responses.

Nearly 2 billion doses of COVID-19 vaccines have been used in Chinese health care populations. The antiviral ability of the inactivated COVID-19 vaccine in the real world and the changes in induced neutralizing antibody (NAb) levels over time are still unclear. Zhang recently reported the level of NAbs induced by inactivated COVID-19 vaccines in the real world [5]. This study was based on serum samples collected from 1335 vaccinated individuals over 18 years of age and found that the NAb positivity rate was 77.3% based on 2 doses of inactivated COVID-19 vaccine [6]. These results were consistent with the currently published phase III clinical efficacy data of the inactivated COVID-19 vaccine [7]. NAb positivity rates peaked at 82%–100% between 10 and 70 days after the second dose of the vaccine and then gradually decreased to 27% after 332 days [8]. Therefore, it was recommended that the third doses be administered within 61 to 70 days after the second dose to induce sustained NAb levels. According to The Technical Guidance on COVID-19 Vaccination (first edition) issued by the National Health and Medical Commission of China, 2 doses of inactivated vaccination were recommended.

In addition, newly published studies have shown that the positivity rate of NAbs is related to age, not sex [9]. After 2 doses of the vaccine, the positivity rate of NAbs among individuals aged 18 to 40 years was considerably higher than that among those aged 41 to 60. Another study found that NAb levels correlated linearly with immunoglobulin M (IgM)/IgG antibodies in recipients [10]. NAbs can still be detected 90 days after the second dose of Moderna’s mRNA vaccine but will inevitably decline over time [11]. Even if the activity of serum NAbs against COVID-19 declined, the vaccine would still trigger a strong B-cell immune response, which will quickly produce targeted NAbs. The vaccine is still effective in response to mutated COVID-19 strains because the current mutated COVID-19 epidemic strains have only mutated at single sites, and a large number of NAbs can still cover the S protein epitope of the virus [12].

The emergence of new COVID-19 variants has made the prevention of COVID-19 difficult, and the NAb titers of the currently marketed vaccines against mutated strains will decrease. To better optimize inactivated vaccine-induced immune response and improve vaccine protection efficiency, this preliminary study was conducted on the factors influencing the production of NAb titers against the inactivated COVID-19 vaccine.

## 2. Materials and methods

### 2.1. Participations

Enrolled in this study were 91 health care volunteers from the Immunology Division of the Laboratory Department of Chongqing General Hospital (3 hospitals) from February to March 2021. Inclusion criteria included being between 30 and 60 years of age with no history of COVID-19 and having a negative PCR test result for severe acute respiratory syndrome coronavirus 2 (SARS-CoV-2). Exclusion criteria included (but were not limited to) having immunosuppressive therapy (including steroids) within the past 6 months, bleeding disorders, asplenia, receipt of any blood products or immunoglobulins within the past 3 months, any confirmed or suspected autoimmune or immunodeficiency disease, metabolic diseases (i.e. hypertension and diabetes mellitus) and a positive PCR test result for SARS-CoV-2. The study protocol containing the full list of eligibility criteria is available online [13]. This study was conducted with the approval of the ethics committee of Chongqing General Hospital (KYS2021-008-01). Informed consent was obtained from all of the individuals included in this study. Clinical and laboratory features, including age, sex, peripheral lymphocyte levels (CD3^+^%, CD3^+^CD4^+^%, CD3^+^CD8^+^%, CD3^+^CD4^+^CD8^+^%, CD19^+^%, CD16^+^CD56^+^%, CD4^+^/CD8^+^), cytokine levels [interleukin (IL)-17F, IL-21, IL-2, IL-4, IL-5, IL-6, IL-8, IL-1β, IL-17A, IL-10, tumor necrosis factor-alpha (TNF-α), tumor necrosis factor-beta (TNF-β), IL-12p70, and interferon-gamma (IFN-γ)], comprehensive metabolic panel (CMP) including glucose, calcium, sodium, potassium, carbon dioxide, chloride, albumin (ALB), total protein (TP), alkaline phosphatase (ALP), alanine transaminase (ALT), and aspartate aminotransferase (AST), blood urea nitrogen (BUN), creatinine, direct bilirubin (DBIL), total bilirubin (TBIL), and urea, were collected for further analysis.

The volunteers received the first vaccination (inactivated SARS-CoV-2 vaccine, Beijing Institute of Biological Products Co., Ltd.) from February 2nd to March 10th, 2021. The second dose was administered 14 days after the first dose. For testing purposes, 5 mL of peripheral venous blood was collected from the antecubital vein in the morning, after fasting, into an ethylenediaminetetraacetic acid (EDTA) anticoagulation tubes. Venous blood was collected for antibody detection at the following times: the first collection was before the first COVID-19 vaccination, and the second collection was before the second COVID-19 vaccination (2 weeks after the first vaccination). The third collection was 1 week after the second COVID-19 vaccination, and the fourth collection was 3 weeks after the second COVID-19 vaccination. All of the collected serum specimens were inactivated in a water bath at 56 °C for 1 h.

The levels of peripheral lymphocytes and cytokines were measured twice; the first time was before the first COVID-19 vaccination, and the second was 1 week after the second COVID-19 vaccination. A CMP and liver and kidney function examinations were conducted 3 weeks after the second COVID-19 vaccination.

### 2.2. Reagents and instruments

First, 2 mL of anticoagulated venous blood was collected from the 4 samples taken from each patient, centrifuged at 2000 × *g* for 15 min and stored at −80 °C until use. The total antibody detection reagent used was InnoDx reagent (magnetic particle chemiluminescence method, batch number: 20210101) with an automatic chemiluminescence immunoanalyzer (Xiamen InnoDx Biotechnology Co., Ltd., Caris 200). The NAb detection reagent used was YHLO reagent (Shenzhen YHLO Biotech Co., Ltd., Reagent, magnetic particle chemiluminescence method, batch number: 20210101) with an automatic chemiluminescence analyzer (iFlash 3000-a). A result for the total antibody signal/cutoff (S/Co) ratio less than 1.00 was considered negative, and a S/Co ratio greater than or equal to 1.00 was considered positive. A concentration of NAbs less than 10.00 AU/mL was regarded as nonreactive, and a concentration greater than or equal to 10.00 AU/mL was considered reactive. Then, 2 mL of anticoagulated venous blood was collected twice to count the absolute number of lymphocyte subsets, which employed a lymphocyte subgroup detection reagent (BD Multitest 6-color TBNK reagent) using a flow cytometer (BD FACSCanto II). Cytokines were analyzed for the serum sample using cytokine assay kits (Weimi Bio-Tech Co., Guangzhou, China; batch number: 20201102) using a BD FACS CantoII flow cytometer (Becton, Dickinson and Company, Franklin Lakes, NJ, US) following the manufacturer’s’ instructions. The kits included 14 types of microbeads with distinct fluorescence intensities and were coated with, respectively, specific antibodies against IL-17F, IL-21, IL-2, IL-4, IL-5, IL-6, IL-8, IL-1β, IL-17A, IL-10, TNF-α, TNF-β, IL-12p70, and IFN-γ. After incubation with the serum sample, the immunocomplex was further combined with phycoerythrin fluorescently labeled detection antibody to form a double-antibody sandwich complex, and the fluorescence intensity of the complex was analyzed using a flow cytometer to quantify the cytokines. Next, 2 mL of EDTA-K2 anticoagulant and 2 mL of nonanticoagulated venous blood were collected for the CMP. A Siemens 2400 automatic biochemical analyzer (Siemens Healthcare, Erlangen, Germany) and supporting reagents were used to measure liver and kidney function, and a Sysmex XE2100 automatic blood cell analyzer (Sysmex Corp., Kobe, Japan) and supporting reagents were used to measure routine blood parameters.

### 2.3. Statistical analysis

The clinical and laboratory features were presented as the mean ± standard deviation (SD). All of the statistical analyses were conducted using R software (R Core Team, R Foundation for Statistical Computing, Vienna, Austria. http://www.R-project.org/). Principal component analysis (PCA) was performed using the R package, ggbiplotof. All of the variables were tested to determine the normality of the distribution before the comparison was conducted, and then, the comparison of the continuous variables (CD16^+^CD56^+^ NK-cell, CD19^+^ B cells, CD4^+^/CD8^+^ T cells, urea, MCV%, IL-2, and IL-6) between group of NAb (+) vs. NAb (−) using parametric (if the data indicated normal distribution, the student t test was used) and nonparametric tests (if the data indicated nonnormal distribution, the Mann-Whitney U test was used) was performed. The dependent variables of the NAb titers (NAbs less than 10.00 AU/mL as nonreactive and NAbs greater than or equal to 10.00 AU/mL as reactive) and independent variables (CD16^+^CD56^+^ NK cells, CD19^+^ B cells, CD4^+^/CD8^+^ T cells, urea, MCV%, IL-2, and IL-6) were defined in the logistic regression analysis, which was employed to compare the successful vaccination effects among the 91 health care volunteers. Some of the plots were generated using GraphPad Prism 8.4. P < 0.05 was considered statistically significant.

## 3. Results

### 3.1. Demographic characters

A total of 91 health care volunteers who were scheduled to receive a complete dose regimen of the inactivated SARS-CoV-2 vaccine were enrolled in the current study. Among them, 40.66% (n = 37) were males (mean age = 38 years; range, 21–59 years), and 59.34% (n = 54) were females (mean age = 44 years; range, 21–57 years). All of the volunteers had undergone cytokine and lymphocyte level tests, a CMP, and liver and kidney function examinations within 2 days before the first vaccination and 1 week after the first vaccination. All of the samples showed normal reference value range results in the biochemical tests before the first vaccination.

### 3.2. Neutralizing antibodies in the healthcare volunteers after COVID-19 vaccination

**Figure 1:**
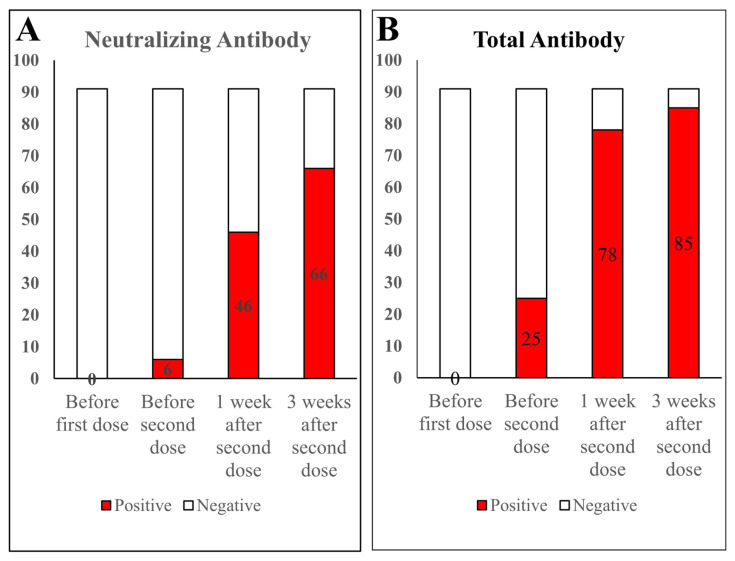
Number of samples that tested positive for antibodies at different time points. **A.** Neutralizing antibody production in 91 samples. **B.** Total antibody production in 91 samples.

All of the volunteers had neutralizing and total antibodies detected at 4 separate time points; within 2 days before the first vaccination, before the second vaccination, one week after the second vaccination, and 3 weeks after the second vaccination. Antibody acquisition after COVID-19 vaccination in the volunteers is shown in Figure 1. The NAb titers gradually increased, and 72.53% (n = 66) of the volunteers had NAbs (Figure 1A). Moreover, 93.4% (n = 85) had total antibodies (Figure 1B).

Then, the volunteers who were positive for NAbs 3 weeks after the second vaccination were defined as having a successful vaccination (effects, n = 66) and those who were negative for NAbs were defined as having a failed vaccination (no effects, n = 25). The univariate logistic regression analysis indicated that 8 variables were associated with the NAb titers after COVID-19 vaccination (Table). However, the multivariate logistic regression analysis demonstrated that only 2 variables correlated with the NAb titers after COVID-19 vaccination, including the urea level (OR = 2.32, 95% CI = 1.08–4.98, p = 0.03) and the increment in CD19^+^ B cells between the second and first vaccinations (OR = 1.96, 95% CI = 1.19–3.705, p = 0.03) (Table).

**Figure 2:**
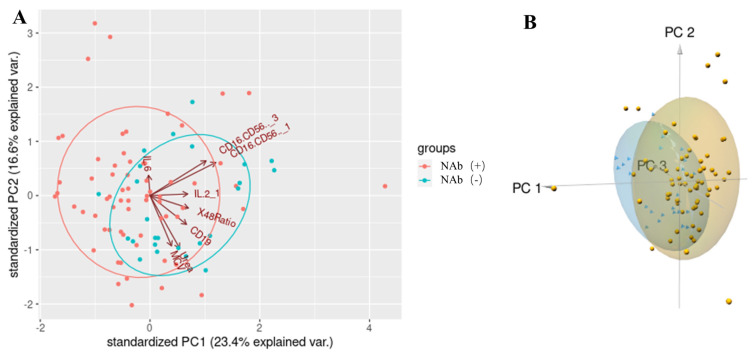
PCA of 8 laboratory features between volunteers with and without NAb titers after COVID-19 vaccination. **A.** Two-dimensional plot of PC1 and PC2. **B.** Three-dimensional plot of PC1, PC2, and PC3.

**Figure 3:**
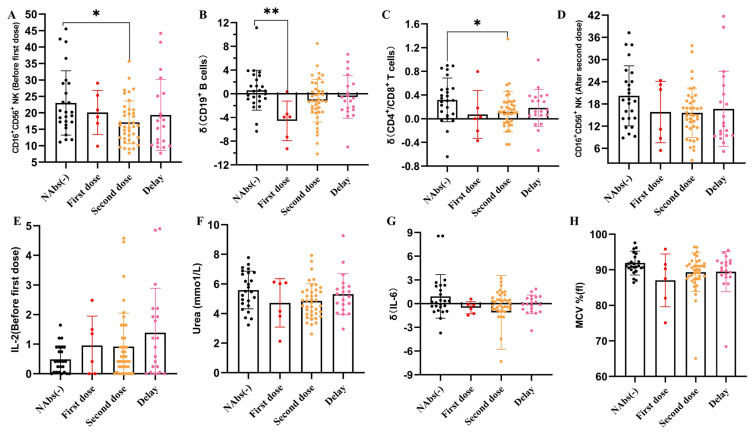
Relationships between NAbs and **A.** CD16^+^CD56^+^ NK cell level before the first dose, **B.** CD19^+^ B cell level, **C.** CD4^+^/CD8^+^ T cell level, **D.** CD16^+^CD56^+^ NK-cell level after the second dose, **E.** IL-2 level before the first dose, **F.** Urea level, **G.** IL-6 level, and **H.** MCV% (H) in the health care volunteers.

PCA effectively distinguished the NAb titers of after COVID-19 vaccination using 8 variables, including the CD16^+^CD56^+^ NK cell level before the first dose, CD16^+^CD56^+^ NK cell level after the second dose, IL-2 level before the first dose, mean corpuscular volume, urea level, and the increment of CD19^+^ B cells between the second and first doses, the increment of CD4^+^/CD8^+^ T cells (also as X48Ratio) between the second and first doses, and the increment of the IL-6 level between the second and first doses in 2-(Figure 2A) or 3-dimensional plots (Figure 2B).

### 3.3. Relationships between the NAbs and laboratory features in the healthcare volunteers

**Figure 4:**
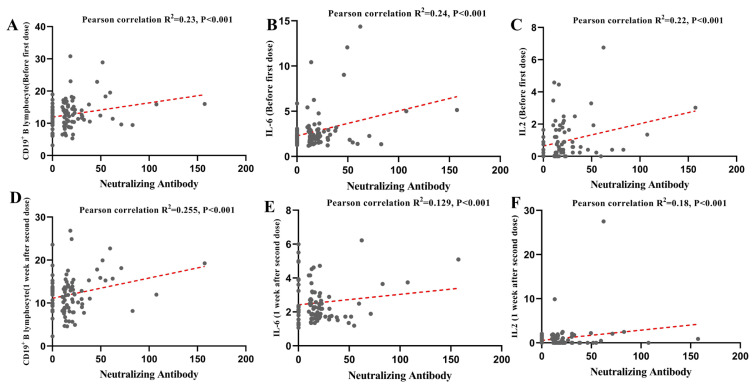
Pearson correlation analysis of the relationships between the NAb concentrations and **A.** CD19^+^ B lymphocyte level before the first dose, **B.** IL-6 level before the first dose, **C.** IL-2 level before the first dose, **D.** CD19^+^ B lymphocyte level after the second dose, **E.** IL-6 level after the second dose, and **F.** IL-2 level after the second dose in the health care volunteers.

Among the successful vaccination volunteers (n = 66), 6 achieved NAbs after the first COVID-19 vaccination, 40 achieved NAbs 1 week after the second COVID-19 vaccination, and 20 achieved NAbs 3 weeks after the second COVID-19 vaccination. Then, the relationships between the NAb titers and the abovementioned 8 laboratory features were examined in the health care volunteers. The correlations between the NAbs and laboratory features are presented in Figure 3. The CD16^+^CD56^+^ NK-cell level before the first dose was higher in volunteers without NAb titers than in those who achieved NAbs 3 weeks after the second COVID-19 vaccination (Figure 3A, p < 0.05). Volunteers who achieved NAbs after the first COVID-19 vaccination had lower levels of CD19^+^ B cells than in those without NAb titers (Figure 3B, p < 0.01). Volunteers who achieved NAbs after the second COVID-19 vaccination had lower levels of CD4^+^/CD8^+^ T cells than those without NAb titers (Figure 3C, p < 0.05). No differences were found between the NAb levels and the other laboratory features (Figures 3D–3H). In addition, the Pearson correlation analysis indicated that 4 variables (CD19^+^ B cell level before and after vaccination, IL-6 and IL-2 level before vaccination) were weakly positively correlated with the concentration of NAbs (Figures 4A–4D), and 2 variables (IL-6 and IL-2 after vaccination) were found no significant correlation with NAbs (Figures 4E and 4F). Therefore, the ratio of CD19^+^ B cells and the levels of IL-2 and IL-6 were positively correlated with the achieved NAbs.

## 4. Discussion

In the current research, 91 health care volunteers achieved NAb titers against the inactivated COVID-19 vaccine. Clinical and laboratory features, such as the cytokine and lymphocyte levels, a CMP, and liver and kidney function examination, were collected as a standard for guiding vaccination. The results showed that the NAb titers produced by the vaccine gradually increased over time, and a success rate of 72.53% was achieved for the inactivated COVID-19 vaccine 3 weeks after the second dose. The univariate and multivariate logistic regression analyses demonstrated that the urea level and the increment of CD19^+^ B cells between the second and first doses were weakly correlated with the NAb titers after COVID-19 vaccination.

COVID-19 pneumonia caused by coronavirus has posed a great threat to human health worldwide. To deal with the fast-spreading epidemic, vaccines are the most effective way to protect people against infection [14, 15]. Presently, 5 types of COVID-19 vaccines are used that employ different techniques, namely, mRNA vaccines, recombinant protein vaccines, virus vector vaccines, DNA vaccines, and inactivated vaccines [16]. Tests from clinical trials have shown that the protection rates of 2 marketed mRNA vaccines, BNT162b2 and mRNA1273, were 95% and 94.1%, respectively [17]. The marketed viral vector vaccines are mainly adenovirus vaccines, including adenovirus type 5, type 26, and chimpanzee adenovirus-vectored vaccines [18]. The chimpanzee adenovirus-vectored COVID-19 vaccine has a protection rate of 70.4%, the type 5 adenovirus-vectored COVID-19 vaccine has a protection rate of 65.7%, and Russia’s type 5 and 26 adenovirus-vectored COVID-19 vaccines have a protection rate of 91.6% [19]. There are many inactivated vaccines and recombinant protein vaccines on the market with different protection rates. The COVID-19 recombinant protein vaccine, NVX-CoV2373, can provide a protection rate of 89.3%, and the protection rates of the inactivated COVID-19 vaccines are 79.34% and 50.38% [20]. Among COVID-19 vaccines, more than 2 billion doses of inactivated vaccines have been administered in Chinese populations. In the current study, a protection rate of 72.53% was achieved for the inactivated COVID-19 vaccine 3 weeks after the second dose. More importantly, 6 volunteers seroconverted to NAbs after the first dose, which means that they had a rapid response to the inactivated COVID-19 vaccine. Clinical trial data has shown that the NAb titers produced by the inactivated COVID-19 vaccines from different sources demonstrated a variety of effects. The NAb titers of the inactivated vaccine CoronaVac was 64 AU/mL, and the NAb titers of 2 other types of inactivated vaccines were 282.7 AU/mL and 247 AU/mL. Moreover, 6 volunteers achieved NAbs after the first dose of COVID-19 vaccine, and the average NAb titre was 13.38 AU/mL. The NAb titers increased to 21.58 AU/mL (n = 46) and 26.79 AU/mL (n = 66) after 1 week and 3 weeks of the second dose, respectively. Moreover, studies have shown that the antibody titers produced by the vaccine continue to decline over time. Research has shown that the NAb response produced by 3 doses is significantly greater than that induced by 2 doses [21]. The NAb and binding antibody responses induced after an interval of 28 days were better than those induced after an interval of 14 days, which may be because the long immunization interval can induce better immune memory responses and rapidly produce stronger antibody immune responses. The results of the current study show that the NAb titers of the inactivated vaccine against COVID-19 gradually increased. However, 25 health care volunteers did not achieve NAbs.

Inactivated COVID-19 vaccine-induced specific humoral immunity is one of the main forces against infection. To explore the response mechanism of COVID-19 infection, especially the specific B-cell immune response, researchers have used high-throughput single-cell sequencing and LIBRA-seq (linking B cell receptors (BCRs) to antigen specificity through sequencing) technology to discover a unique subset of activated memory B cells (CD11c^high^ CD95^high^), which have high levels of antigen markers [22]. COVID-19 B-cell responses have potential value. After analyzing the BCR spectrum, researchers combined LIBRA-seq to efficiently screen out the coronavirus antigen-specific antibody and obtained coronavirus receptor-binding domain (RBD)-induced highly active NAbs. Therefore, consistent with previous studies, the ratio of CD19^+^ B cells determined the reaction after COVID-19 vaccination, and a high percentage of CD19^+^ B cells was positively correlated with the concentration of NAbs. In addition, the current study showed that IL-6 cytokines in the volunteers were correlated with the production of NAbs [23]. IL-6 is a cytokine expressed at the earliest stage of tissue injury and infection that induces the synthesis of C-reactive protein (CRP) and fibrinogen in the acute phase response. The elevated levels of IL-6 in the volunteers may have been caused by the direct or indirect activation of IL-6 expression by bacteria or viruses, or it may have been secondary to a hyperinflammatory response similar to macrophage activation syndrome after viral infection, which means that the body is in a state of immune response [24]. The Diagnosis and Treatment Protocol for Novel Coronavirus Pneumonia (Trial Version 7), issued by the National Health and Medical Commission of China, pointed out that clinical early warning indicators include elevated levels of IL-6 [25]. The possible mechanism for the elevation of IL-6 is inducing immune cells that produce a large number of inflammatory factors (such as granulocyte-macrophages). Hasan et al. pointed out that cytokine levels measured serially, including IL-6 at different sampling times, provided a more precise and accurate estimate for the outcome of COVID-19 patients [26]. A study on the levels of inflammatory factors in the early stage of septic shock showed that the level of IL-6 in patients with septic shock was significantly higher than that in nonseptic patients, and IL-6 was an independent risk factor for septic shock. However, the IL-6 level cannot be used to distinguish between bacterial infection or viral infection, and some noninfectious factors, such as trauma and surgery, can also cause an increase in the IL-6 level. More interestingly, the current investigation showed that the IL-6 level can be used to assess the production of NAbs after COVID-19 vaccination. By detecting the levels of IL-6, it is possible to identify the success rate of COVID-19 vaccination. However, further research on the pathophysiological process and mechanism of occurrence and development of COVID-19 immunity is required. Recently, other cell types were also mentioned regarding the induction of trained immunity following a single dose of the ChAdOx1nCoV-19 vaccine and SW0123 vaccination effectively suppressed SARS-CoV-2-induced inflammatory responses by inhibiting the recruitment of proinflammatory macrophages and increasing the frequency of polymorphonuclear myeloid-derived suppressor cells using high-resolution single-cell analysis [27, 28]. To reveal more mechanisms about COVID-19 vaccination, these conclusions need to be further clarified.

In summary, the results herein showed that the NAb titers of inactivated vaccines were positively associated with the ratio of CD19^+^ B cells and IL-6 and IL-2 levels, which could provide clinical guidance for inactivated SARS-CoV-2 vaccination. However, this study included only a young and low-risk population with a short follow-up period before the emergence of viral variants. Further data are needed from more volunteers on the performance of COVID-19 to demonstrate the efficacy of the vaccine against the variants of concern and the duration of protection in populations including older adults, adolescents, and children, and individuals with specific chronic diseases.

## Figures and Tables

**Table:** Univariate and multivariate analyses of the factors correlated with the level of neutralizing antibodies.

Variables	Univariate analysis			Multivariate analysis		
	HR	95% CI	p-value	HR	95% CI	p-value
CD16+CD56+ NK (before the first dose)	0.94	0.89–0.99	0.02	0.93	0.83–1.03	0.15
CD16+CD56+ NK (after the second dose)	0.94	0.89–0.99	0.03	0.96	0.87–1.05	0.34
IL-2 (before the first dose)	2.09	1–4.33	0.05	2.23	0.8–6.26	0.13
MCV % (fl)	0.86	0.75–0.98	0.02	0.86	0.73–1.02	0.08
Urea (mmo1/L)	0.69	0.48–1	0.05	2.32	1.08–4.98	0.03
δ (CD19+ B cells)	0.86	0.75–0.99	0.03	1.96	1.19–3.705	0.03
δ (CD4+/CD8+ T cells)	0.21	0.05–0.86	0.03	0.27	0.04–1.68	0.16
δ (IL-6)	0.75	0.57–0.98	0.04	0.68	0.45–1.02	0.06
